# Gsk3 Signalling and Redox Status in Bipolar Disorder: Evidence from Lithium Efficacy

**DOI:** 10.1155/2016/3030547

**Published:** 2016-08-18

**Authors:** Antonina Luca, Carmela Calandra, Maria Luca

**Affiliations:** ^1^Department of Medical and Surgical Sciences and Advanced Technologies, University Hospital Policlinico-Vittorio Emanuele, Santa Sofia Street 78, Catania, 95100 Sicily, Italy; ^2^Psychiatry Unit, Department of Medical and Surgical Sciences and Advanced Technologies, University Hospital Policlinico-Vittorio Emanuele, Santa Sofia Street 78, Catania, 95100 Sicily, Italy

## Abstract

*Objective*. To discuss the link between glycogen synthase kinase-3 (GSK3) and the main biological alterations demonstrated in bipolar disorder (BD), with special attention to the redox status and the evidence supporting the efficacy of lithium (a GSK3 inhibitor) in the treatment of BD.* Methods*. A literature research on the discussed topics, using Pubmed and Google Scholar, has been conducted. Moreover, a manual selection of interesting references from the identified articles has been performed.* Results*. The main biological alterations of BD, pertaining to inflammation, oxidative stress, membrane ion channels, and circadian system, seem to be intertwined. The dysfunction of the GSK3 signalling pathway is involved in* all* the aforementioned “biological causes” of BD. In a complex scenario, it can be seen as the common denominator linking them all. Lithium inhibition of GSK3 could, at least in part, explain its positive effect on these biological dysfunctions and its superiority in terms of clinical efficacy.* Conclusions*. Deepening the knowledge on the molecular bases of BD is fundamental to identifying the biochemical pathways that must be targeted in order to provide patients with increasingly effective therapeutic tools against an invalidating disorder such as BD.

## 1. Introduction

GSK3 is a Ser/Thr kinase originally identified as an enzyme highly specific for glycogen synthase [1]. However, the regulation of glucose metabolism is only one of the incredibly complex functions of this unique kinase. It has been in fact demonstrated that GSK3 plays a central role in cellular signalling, regulation of cell proliferation, and neurodevelopment [1].

A dysregulation of GSK3, usually in terms of hyperactivation, has been called into question for the pathophysiology of several diseases, such as diabetes mellitus, neurodegenerative disorders (e.g., Alzheimer's disease), cancer, and psychiatric disorders, BD in particular [2]. As far as the latter is concerned, recent researches suggest that oxidative stress (OS) is crucially involved in its pathogenesis [3]. OS seems to be strictly linked to GSK3, since many studies have reported that the kinase is sensitive to redox homeostasis and that its signalling is involved in the occurrence of the OS-related damage [4]. Despite the increasing interest on the pathogenesis of BD, there is still a need of further insights. In fact, BD is a complex, rather frequent disorder that has been related to different biological substrates (e.g., altered circadian system) explaining only certain aspects of the heterogeneous clinical features [5–7]. It is challenging to identify a single, shared basis accounting for all the main biological alterations reported in BD in the last years. In this regard, GSK3 pathway seems to be a promising candidate.

Some specific questions arise pertaining to the role of GSK3 in BD: (a) how is GSK3 involved in the pathogenesis of BD? (b) Can the involvement of GSK3 explain all the complex biological alterations demonstrated in BD, OS in particular? (c) Does the involvement of GSK3 have clinical implications? (d) Is GSK3 the right target for the psychopharmacological therapies? (e) Is GSK3 the key of lithium efficacy?

In the attempt to answer to these questions, this review focuses on the link between GSK3 and the main biological alterations demonstrated in BD, with special attention to the redox status, also taking into consideration the evidence supporting the efficacy of lithium (a GSK3 inhibitor) in the treatment of this psychiatric disorder.

## 2. Materials and Methods

### 2.1. Research Strategy

A literature research on the discussed topics, using Pubmed and Google Scholar, has been conducted. Moreover, a manual selection of interesting references from the identified articles has been performed.

#### 2.1.1. GSK3: General Information

**Figure 1:**
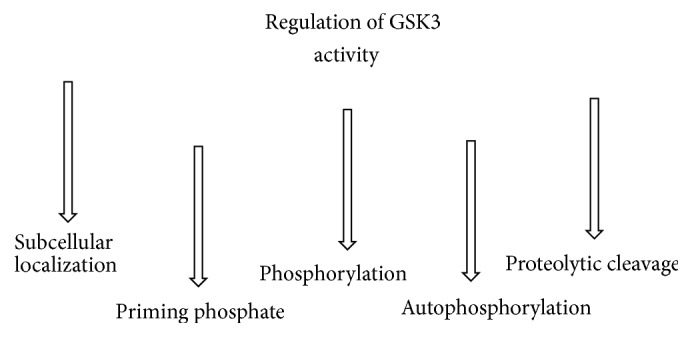
The figure shows the main regulatory mechanisms of GSK3 activity. GSK3: glycogen synthase kinase 3.

It is highly conserved during mammalian evolution. Two GSK3 isoforms, *α* (51 kDa) and *β* (47 kDa), practically identical in terms of amino acid sequence, are known. GSK32 is a variant of GSK3*β* discovered in 2002 containing a 13-amino acid residue [1, 8]. Many of the substrates of GSK3 need a “priming phosphate” (a phosphorylated Ser/Thr residue) triggering the phosphorylation process, the latter usually determining the inactivation of the substrate. GSK3 is constitutively active, but its activity is strictly regulated through multiple ways. The need of a priming phosphate represents itself as a form of regulation. In addition, GSK3 is susceptible to serine (inhibitory) and tyrosine (stimulatory) phosphorylation by other kinases and even by autophosphorylation [9]. Protein kinase B/Akt and Wnt signalling pathway are two renowned inhibitors of GSK3 [1, 10]. Tyrosine kinase Pyk2 is an activator of GSK3. The specific residues targeted by the inhibitory or activatory kinases can change according to the isoform considered [11]. The subcellular localization of GSK3 is another regulator of its activity: nuclear GSK3, for example, is specifically involved in the regulation of transcription factors. Moreover, the removal of specific fragments (e.g., serines 9/21) from the N-terminal region of GSK3 (proteolytic cleavage) is a recently reported activatory mechanism [12] (Figure 1). GSK3 is widely distributed in the human body and nearly 100 proteins have been identified as its substrates. However, it is highly represented in the brain [13]. In fact, GSK3 is involved in several aspects of the neuronal development [14]. More specifically, it regulates neuronal differentiation (from polarization to migration), axonal growth [15, 16], survival of neuronal cells, synaptic transmission [17, 18], neurotransmitter release [18], regulation of ions channels, and neuronal cell cycle [19]. The importance of GSK3 is confirmed by its extremely relevant targets: Wnt system, insulin pathway, nuclear factor kappa beta (NF-*κ*B), cAMP response element-binding protein (CREB), tau [2, 13], heat shock factor-1, nuclear factor of activated T cells, and beta-catenin [9], just to name a few. It goes without saying that GSK3 plays a fundamental role in crucial physiological processes, among which apoptosis, cellular differentiation and proliferation, inflammation, glucose metabolism, and stem-cell renewal [2].

#### 2.1.2. GSK3 and Oxidative Stress

By redox homeostasis we mean the maintenance of the balance between reactive oxygen species (ROS) production and elimination. When it fails, the production of reactive species exceeds their elimination and OS occurs with detrimental effects on DNA, lipids, and proteins [20]. Since mitochondria are great producers of ROS, their dysfunction relates to OS in a vicious cycle where the damaged mitochondria produce more ROS which, in turn, cause them further damage [21].

**Figure 2:**
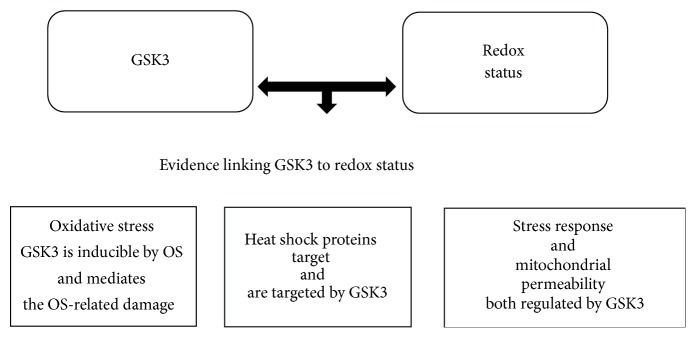
The strict link between GSK3 and redox status is explained according to the evidence. GSK3: glycogen synthase kinase 3; OS: oxidative stress.

Growing evidence suggests a role of GSK3 in the regulation of the mitochondrial function. In fact, GSK3 has been found to directly control the mitochondrial permeability transition in cultured renal tubular epithelial cells through the phosphorylation of its targets [4]. Moreover, it has been demonstrated that GSK3 can translocate from the cytosol to the mitochondria through a fascinating mechanism of recognition of an N-terminal domain [22]. GSK3 is involved in the response to stress and the link between this kinase and OS is so strong that some authors have defined GSK3 as “redox-sensitive enzyme” [23]. The inhibition of GSK3 has been found to be protective against the OS-related cellular loss [4] and to protect the mitochondrial function [24, 25]. GSK3 is inducible by OS [26] and represents a pathway involved in the stress response [27, 28]. Moreover, GSK3 is the target of the heat shock proteins (Hsp_s_), molecules regulating the redox status, and the protein folding. At the same time, Hsp_s_ present sites targeted by GSK3 [29–31]. In addition, the redox status interferes with GSK3 activity; in fact, 4-hydroxynonenal, a major product of membrane lipid peroxidation, is a powerful inhibitor of GSk3 via the phosphorylation in Ser9 [23]. The phosphorylation of Ser9 is performed by anticancer drugs and relates to the enhancement of the antioxidant defences, such as heme-oxygenase 1, in the cells [32]. GSK3 usually mediates the negative effects of OS [4, 33] through, for example, the inhibition of transcription factors, thus leading to massive cell loss [34]. Hsp90 contributes to the stability of GSK3 and protects it from degradation. The decrement of tau phosphorylation due to Hsp90 inhibitors has been related to a reduction in the levels of GSK3 [30] (Figure 2).

Furthermore, GSK3 takes part in the complex regulatory mechanisms of cell survival. Its crucial role in mitochondrial permeability, its cross talk with a huge number of other signalling pathways, and the multiple ways controlling its activity make GSK3 an extremely fascinating kinase with effects that are sometimes difficult to understand [35, 36]. This is why GSK3 can exert both prosurvival and prodeath effects and its inhibition can result in different outcomes according to the cellular type. From a general point of view, the acute stimulation of GSK3 could favor adaptive cellular mechanisms, while its chronic overexpression could promote massive cell loss or proliferation according to the tissues involved [35]. Simplistically, it can be stated that the overexpression of GSK3 is generally considered a key step in the pathogenesis of several disorders, such as Alzheimer's disease, cancer, diabetes, and BD [8], mostly because of its prooxidative and proinflammatory properties [34, 37].

#### 2.1.3. The “Biological Causes” of BD

BD is a common and invalidating psychiatric disorder characterized by recurrent manic and depressive symptoms. This disorder presents a high rate of heritability and cannot be defined as a “brain disorder” but as a systemic disease. Many “biological causes” of BD have been studied in recent years. In particular, the most studied abnormalities pertain to inflammation (altered levels of cytokines and altered apoptosis regulation), oxidative stress (mitochondrial dysfunctions and altered endoplasmic reticulum (ER) stress response), membrane ion channels, and circadian system [5–7].

(*1) Inflammation*. Several reports have highlighted a higher concentration of interleukin (IL) 4 and 2-R as well as tumor necrosis factor (TNF) alpha in patients with BD. The altered inflammatory status has been found in euthymic phases, but it is enhanced in case of a depressive/manic episode [38].

It has been stated that BD could be considered as a “multisystem inflammatory disease.” In fact, after controlling for life-style habits and adverse effects of pharmacological treatments, BD presents high rates of comorbidity with cardiovascular disorders. Proinflammatory cytokines are highly expressed from the occurrence of BD to the late stage of the disease, during manic and depressive episodes. It is well known that inflammation represents the trigger for many cardiovascular risk factors, such as atherosclerosis and diabetes [39]. Inflammation affects mood, since it activates the degradation of both tryptophan and serotonin and relates to behaviours favoring suicide, such as aggression, impulsivity, and depression [39, 40]. Moreover, the proinflammatory status decreases the expression of the brain-derived neurotrophic factor (BDNF) and induces OS and nitrosative stress, thus contributing to the neural loss [39]. The link between inflammation and OS has been recently related to the nod-like receptor pyrin domain-containing 3 (NLRP3), an inflammatory redox sensor whose activation has been associated with the production of ROS [41]. In addition, the expression of nitric oxide (NO), typical of the inflammatory states, can facilitate the occurrence of OS, thus leading to cellular damage, particularly in the brain. NF-*κ*B, a family of proteins crucially involved in inflammation, plays a central role in limiting the NO-related apoptotic processes; NO itself enhances its activity [42].

**Figure 3:**
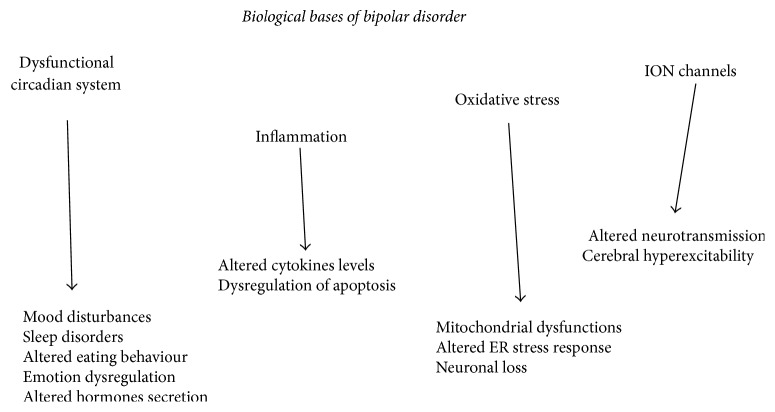
The biological alterations reported in bipolar disorder are shown, as well as their consequences. ER: endoplasmic reticulum.

Neuroprogression, accounting for the neurodegenerative processes and the worsening of the clinical features occurring over the course of BD (e.g., shortening of symptom-free intervals, reduced response to therapy, and cognitive decline) has been linked to stage-dependent inflammatory and redox status [43]. In fact, the expression of the anti-inflammatory IL-10 decreases over time, decreeing the failure of the compensatory mechanisms against inflammation. On the contrary, the antioxidant defences such as glutathione are expressed as a tardive attempt to protect the cells from further OS-related damage. Unfortunately, the antioxidant agents seem to be ineffective, since BD is sadly characterized by various evidences of oxidative damage [44].

(*2) OS*. A dysfunction in the mitochondria complexes has been demonstrated in bipolar patients [45]. More specifically, the mitochondrial complex I subunits seem to be particularly involved [41, 45]. In fact, a downregulation of this system, and the consequential overproduction of ROS, has been variously reported [41, 46]. In addition, an impaired endoplasmic reticulum (ER) stress response, negatively affecting neurodevelopment and neuroplasticity (it is worth pondering over that ER is involved in the regulation of protein folding), has been identified as one of the multiple biological bases of BD [47]. The investigation of the expression of ER stress-related genes (such as CHOP and calreticulin) on lymphoblastoid cells derived from bipolar patients showed an altered ER stress response [47].

(*3) Ion Channels and Circadian System*. From genome-wide association studies, it is known that many of the genes related to BD regulate the structure and the activity of ion channels (e.g., CACNA1C for calcium channels and KCNQ2 for potassium channels) that are involved in neurotransmission, neuroplasticity, emotional processing, and cognition [18, 48, 49]. Once again, OS is called into question, since mitochondria and ER regulate the intracellular Ca^2+^ signalling [50]. As previously stated, an alteration of the circadian system has been revealed in BD, with different functional profiles according to the current episode of mood alteration [51–53]. The headquarters of the circadian system is the suprachiasmatic nucleus (SCN), considered as a pacemaker [54]. Several circadian clock genes (ARNTL, CLOCK, CRY2, CSNK1epsilon, DBP, NPAS2, PER1, PER2, and PER3), encoding for proteins involved in the generation of the internal rhythm, have been linked to BD [54, 55]. OS is involved in this case, too: the disruption of the circadian system has been in fact related to an increased lipid peroxidation in a sample of female bipolar patients [56]. From a clinical point of view, it is apparent that sleep disorders not only are frequent but represent core symptoms in BD, no matter the phases (depression, mania, and euthymia) [52]. Sleep deprivation, light therapy, and the melatonergic agonist agomelatine are some of the therapeutic tools for mood disorders that confirm the attention drawn to the circadian system [57]. The latter modulates, apart from sleep and hormone secretion [58], emotion regulation [51], eating behaviour [59], and mood [51]; all of these areas can be variously affected in a patient with bipolar disorder [52, 57, 60, 61] (Figure 3).

#### 2.1.4. GSK3 and the “Biological Causes” of BD

GSK3 is involved in all the complex biological alterations of BD (inflammation, oxidative stress, membrane ion channels, and circadian system).

(*1) Inflammation*. GSK3 is known to exert a complex regulatory activity on the inflammatory and apoptotic processes, also according to the cellular localization [62]. Generally speaking, GSK3 can be considered as a proinflammatory molecule, stimulating the production of various inflammatory cytokines and tumor necrosis factors and inhibiting the production of anti-inflammatory agents, such as IL-10. The inhibition of GSK3 has been in fact proven to be beneficial in inflammatory conditions [37].

GSK3 is involved in neuroinflammation, since it enhances the inflammatory response induced by the microglia activation through the activation of TNF alpha [63]. GSK3 is usually seen as a powerful stimulator of apoptosis, but it can exert both proapoptotic and antiapoptotic effects [36] according to the cellular localization (cytosolic GSK3 seems to promote cell survival), the cell type, and the pathways involved [36]. This peculiarity can be explained by its differential regulatory activity on two apoptotic processes: it promotes the mitochondrial intrinsic apoptotic pathway while inhibiting the extrinsic apoptotic signalling pathway. The first is activated by cell damage, and the latter depends on a receptor-mediated mechanism (ligands stimulating death receptors on the cellular surface). This is why the inhibition of GSK3 can result in apparently paradoxical effects on the cell fate [36, 64].

(*2) OS*. As previously stated, GSK3 is a modulator of the mitochondrial functions [65]. In particular, it regulates the mitochondrial permeability transition (MPT) pore. The exposure to OS leads to mitochondrial dysfunction, massive opening of the MPT pore, and cell death. All these detrimental effects have been related to the redox-sensitive activation of GSK3 [4]. For example, the kinase mediates the mitochondrial dysfunction and the dopaminergic cell death caused by 1-methyl-4-phenylpyridinium iodide (MPP^+^), an inhibitor of the mitochondrial complex I [24]. While the inhibition of GSK3 is a promising therapeutic tool against cancer, the chemotherapeutic induction of mitochondrial stress, leading to GSK3 activation, enhances the MPT pore opening, thus promoting tumor cell death [66]. This further supports the fact that GSK3 can show both negative and positive effects. As an example, GSK3 from* Arabidopsis thaliana* has been proved to favor the resistance to salt stress through the activation of Glc-6-phosphate dehydrogenase, which guarantees the redox balance [67].

On the other hand, resveratrol, an antioxidant contained in grapes, protects the cells from oxidative damage, apoptosis, glutathione depletion, and mitochondrial dysfunction. Its antioxidants properties have been in part related to the increased inhibitory phosphorylation of GSK3 downstream of the AMP-activated protein kinase [68]. In addition, the activation of the powerful antioxidant heme-oxygenase 1 has been related to phosphatidylinositol-3 kinases (PI3K)/Akt signalling, involved in the inhibition of GSK3. It is interesting to notice that lithium is a PI3K activator [69]. The PI3K-Akt-GSK3 signalling protects against the GSK3-mediated oxidative damage (massive opening of MPT and ROS production) occurring as a consequence of GSK3 translocation to the mitochondria [70]. A prompt activation of this pathway has been demonstrated to exert protective effects against iron-induced OS in hippocampal neurons [71]. Unfortunately, the considerable changes in cell functioning related to OS can interfere with the efficiency of the compensatory mechanisms. As an example, the powerful oxidant hydrogen peroxide paradoxically increases the inhibitory phosphorylation of GSK3. This would be useful to avoid further damage. Why is, then, the activity of GSK3 increased in the presence of OS, thus favoring the progression of oxidative damage? The answer could lay in the transient increase in Ca^2+^ intracellular concentrations caused by the peroxide. As a result, calpain is activated. This molecule is responsible for the cleavage of GSK3 into two fragments with the loss of the inhibitory domain. The cleavage counteracts the attempts to inhibit GSK3 activity. Hence, the activation of GSK3 by oxidants can be reconducted to the activation of calpain [72]. It is interesting to notice that lithium is capable of inhibiting both full-length and cleaved GSK3 [73]. Some authors have proposed a model explaining the proinflammatory and prooxidative condition characterizing BD as associated with a disruption of the blood-brain barrier integrity. The brain could be then exposed to cytokines and ROS and the activation of microglial cells could further negatively affect the neuronal functions [74]. In this regard, it is noteworthy that the beta-catenin mediated inhibition of GSK3 has been associated with an increased expression of p-glycoprotein, an efflux transporter, in brain endothelial cells [75]. Neuroinflammation has been associated with the activation of GSK3, which alters the barrier properties of the brain through the stimulation of the inflammatory response in the vascular endothelium through the stimulation, for example, of the vascular cell adhesion molecule-1 [76]. The activation of GSK3 can be due to OS and to various other conditions, which are not independent from OS, such as hypoxia, glutamate excitotoxicity, DNA damage, and mitochondrial toxins. The consequence of GSK3 activation lays in apoptosis and many GSK3-mediated apoptotic processes can be reconducted to its effects on mitochondria [77]. In vitro studies on human lens epithelial cells have demonstrated that the inhibition of GSK3 protects against oxidative stress. The cells have been exposed to GSK3 inhibitors and placed, before and after treatment, in a medium containing fluorogenic indicators of oxidative damage. After exposure to GSK3 inhibitors, the cells showed an increased mitochondrial membrane potential, a higher expression of glutathione, and lower markers of apoptosis [78]. Moreover, GSK3 is a regulator of ER stress response. It has been demonstrated that this enzyme enhances the expression of the death inducing the transcription factor CHOP in neural cells after ER stress; the latter often occurs when there is a cellular accumulation of misfolded proteins, as in neurodegenerative disorders [29].

(*3) Ion Channels and Circadian System*. Considering that phosphorylation is a crucial step for the regulation of ion channels [79], it is easy to imagine how GSK3, being a kinase with a huge number of targets, could be involved in the modulation of calcium, sodium, and potassium channels [18]. The impact of GSK3 on neurotransmission is yet unclear, but it is known that it interferes with the synaptic vesicle fusion since its targets are crucially involved in the process of neurotransmitter release [17, 80]. More specifically, GSK3 is responsible for the phosphorylation of an intracellular loop of a P/Q-type voltage-dependent calcium channel, thus negatively affecting the channel activity. In vitro studies allowed to demonstrate that the overexpression of wild type GSK3*β* caused a reduction of neuronal calcium influx [17]. This kinase affects excitability also regulating the highly complex mechanism of the formation of the sodium channel complex favoring the assembly of proteins [81]. The GSK3-dependent phosphorylation of the voltage-gated potassium channel KCNQ2 results in a reduced channel activity [48]. The activity of GSK3 is also extended to the receptor channels, such as those of* N*-methyl-D-aspartate. The inhibition of GSK3 leads to a reduction of the ionic and synaptic current related to this channel [82]. As far as the circadian system is concerned, the importance of GSK3 in its regulation can be deduced by the fact that it is listed among the clock genes, related to BD [54]. GSK3-*β* gene is, in fact, the mammalian ortholog of the* Drosophila* gene SHAGGY encoding for a serine/threonine kinase [83]. In studies conducted on mice, it has been demonstrated that GSK3 phosphorylates at least 5 core clock proteins and shows a circadian oscillation in the phosphorylation state (inactivation) of the *β* isoform [83, 84]. GSK3 interacts with PER2 [83] and this interaction seems to be fundamental to the regulation of the periodicity of the endogenous clock mechanism; in fact, the inhibition of GSK3 leads to a certain delay in the mPER2 transcription. Hence, some authors have proposed GSK3 as a candidate mammalian “core” clock gene [85].

#### 2.1.5. GSK3 in BD: Clinical Aspects

From a clinical point of view, GSK3 has been linked to BD from the evidence that mood stabilizers are GSK3 inhibitors. It has been rightfully suggested to avoid taking for granted that GSK3 is hyperactive in BD since its inhibition is useful in terms of pharmacotherapy. In fact, the alteration could pertain to the upstream or downstream signals linked to GSK3 and the inhibitory agents could reestablish the balance [77]. However, a higher activity of GSK3 has been demonstrated in bipolar patients [86]. Higher levels of both GSK3 isoforms have been reported among patients experiencing a manic episode compared to healthy subjects. After treatment, the levels did not change, but the inhibitory serine-phosphorylation of GSK3 increased [86]. Mice overexpressing GSK3 show a decreased rod b-wave amplitude at *V*_max_ in the electroretinogram, that is, a biological endophenotype in young offspring at high genetic risk for schizophrenia and bipolar disorder [87]. Recent studies reported a dysregulated activity of GSK3, in terms of hyperactivation, due to a lower serine inhibitory phosphorylation among bipolar patients [88]. Hence, more than the overexpression, the dysregulated activity seems to be fundamental in mood disorders, also considering that while the GSK3 activity is finely regulated, its expression is rather stable [88].

Genetic studies further demonstrate the importance of GSK3 in BD.

A single nucleotide polymorphism (−50 T/C) falling into the effective promoter region (nt −171 to +29) of the gene of GSK3*β* has been identified and it has been reported that homozygotes for the wild variant (T/T) show an earlier age at onset of BD than carriers of the mutant allele [89]. In addition, the less active GSK3*β* promoter gene variants relate to milder clinical features of BD; moreover, in a sample of patients the less active GSK3-*β*  rs334558^*∗*^C gene-promoter variants and the long-term administration of lithium were associated with an increase of white matter in several brain regions [90]. Higher frequencies of the C:A haplotype and lower frequencies of the A:C haplotype at the GSK3*β* gene (rs1732170:rs11921360) have been related to a higher risk of suicidal behaviour in bipolar patients [91]. It is worthy of consideration that GSK3 is a regulator of both polarities of mood, since the altered inhibitory serine-phosphorylation of GSK3 is involved in manic-like as well as depressive-like behaviour in mice [92]. Hence, massive evidence supports the central role of GSK3 in BD, as confirmed by the efficacy of lithium, a GSK3 inhibitor, in the treatment of this invalidating psychiatric disorder (see below).

#### 2.1.6. GSK3 in BD: Evidence from Lithium Efficacy

After decades from the first reports of its efficacy [93, 94], lithium still remains the gold standard in the treatment of BD, being efficacious in treating manic and depressive symptoms, in preventing recurrent episodes of mood alteration, and in reducing the suicide risk [95]. It is an alkali metal used under the form of a cationic salt Li^+^ in association with carbonate or citrate [96].

The inhibition of GSK3 has been investigated in the last years as an important target mediating the therapeutic effects of this peculiar drug. Apart from the direct inhibition of GSK3, most probably consisting in competing for a magnesium-binding site within GSK3*β* [96, 97], lithium has indirect inhibitory effects through the activation of the GSK3 inhibitor Akt; as a result, the inhibitory serine-phosphorylation of GSK3 is increased [96]. The latter mechanism has been demonstrated by assessing the level of serine-9 phosphorylated GSK3*β* in platelets of bipolar patients. After treatment with lithium, the level was higher and related to the clinical improvement [98]. Many effects of lithium can be reconducted to its inhibitory action on GSK3.

**Figure 4:**
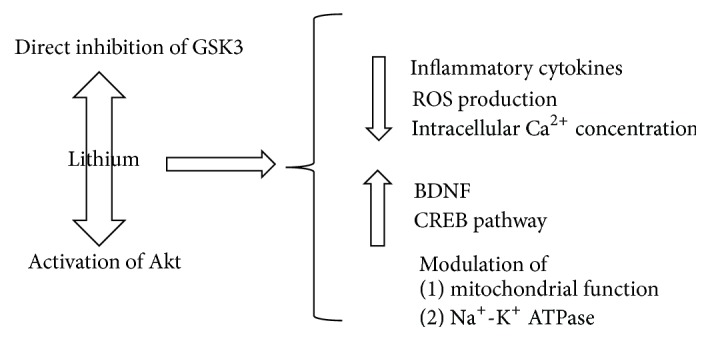
The main activities exerted by lithium, largely reconducted to GSK3 inhibition are here summarized. GSK3: glycogen synthase kinase 3; ROS: reactive oxygen species; BDNF: brain-derived neurotrophic factor.

All of the four previously mentioned “biological causes” of BD (inflammation, oxidative stress, membrane ion channels dysfunction, and altered circadian system) are antagonized by lithium, as described below.

(*1) Inflammation*. Lithium has got anti-inflammatory (significant reduction of IL-6, IL-1*β*, and TNF*α*) and antiapoptotic properties that have been related to its inhibition of GSK3 [26, 40]. In fact, it is considered as a neuroprotective drug and it has been proposed as a potential therapeutic tool against neurodegenerative disorders [99]. Lithium enhances the expression of the BDNF [100, 101], that has been found to be lower in bipolar patients, in both depressive and manic episodes and is considered as a biomarker for BD [102, 103]. The crosstalk between BDNF and the Wnt pathway, which is an important step for the BDNF-related stimulation of the neuronal growth, finds in GSK3 its key mediator. In addition, BDNF indirectly regulates GSK3 activity through the activation of its inhibitor Akt [88].

(*2) OS*. It has been reported that lithium is an antioxidant, as demonstrated by its effect on OS parameters. In particular, lithium reduces the superoxide dismutase and catalase ratio, thus reducing OS. In addition, it exerts a protective role against ROS production, DNA damage, and lipid peroxidation [104, 105]. In addition, lithium seems to exert positive effects on mitochondrial dysfunctions and ER stress response [106, 107]. The inhibition of GSK3 due to lithium administration has been found to be responsible for the resistance to OS of murine hippocampal neuronal cells [108].

(*3) Ion Channels and Circadian System*. A dysregulation in the homeostasis of the ion channels has been demonstrated in BD. In particular, bipolar patients have been found to present an impairment in the Na^+^-K^+^ ATPase [109], which allows the exit of three sodium ions for two potassium ions. The Na^+^/Ca^2+^ exchanger activity depends on the pump. In fact, it allows the entry of Ca^2+^ inside the cell in the presence of high concentrations of Na^+^. Hence, the failure of the Na^+^-K^+^ ATPase leads to intracellular K^+^ depletion and Na^+^ accumulation. As a result, the Na^+^/Ca^2+^ exchanger begins to pump Ca^2+^, thus enhancing the cellular excitability [110, 111]. The hypothesis of cellular hyperexcitability in BD is supported by the finding of higher intracellular Ca^2+^ concentration in platelets of patients compared to controls [112, 113]. Lithium is a powerful regulator of cortical excitability [114]. It modulates the Na^+^-K^+^ ATPase and has a peculiar mechanism of action: it enters the cell through the sodium channel and suppresses the outward membrane current [114]. Lithium also regulates the expression of the isoforms of voltage-dependent Na^+^ channels and part of this activity has been linked to the inhibition of GSK3 [115]. Moreover, lithium reduces the intracellular Ca^2+^ concentrations, blocking the metabolism of the intracellular second messenger inositol-1,4,5-trisphosphate and antagonizing the N-methyl-d-aspartate receptors [116]. It is also fascinating that a Li^+^/Ca^2+^ exchange exists [117]. In addition, lithium affects the regulatory mechanism of calcium storage in the ER performed by the sarcoplasmic/endoplasmic reticulum Ca^2+^-ATPases (SERCAs); the lithium-related inhibition of GSK3 has been shown to determine an increased expression of SERCA2a [118]. Moreover, it has been proposed that a part of lithium efficacy could be due to the block of the GSK3-related phosphorylation of the potassium channel KCNQ2 [48]. As far as the circadian system is concerned, treatment with lithium results in a prolongation of the period of firing rate in the neurons. The effect on the pace-making properties of the cells finds an explanation in the induction of a phase delay in mPer2 transcription consequential to GSK3 inhibition [85] (Figure 4).

#### 2.1.7. GSK3 and OS in BD: What about Lithium

Since GSK3 is so deeply involved in the pathogenesis of BD, it is not surprisingly that its inhibition contributes to the therapeutic actions of lithium, other mood stabilizers, and even electroconvulsive therapy [77]. The importance of GSK3 on lithium efficacy has been demonstrated in genetic studies, too. In fact, GSK3 variants seem to affect the risk of BD, age of onset in females, and response to lithium [119]. The haplotype 1 (T-A) is predictive of a higher response to treatment, while haplotype 2 (C-A) is related to a lower response [120]. Despite the fact that lithium is an “old” drug, its mechanism of action is not deeply understood, and, apart from GSK3, the actions on Na^+^-K^+^ ATPase and on CREB have been identified as crucial steps determining the therapeutic effect [121]. Ouabain, a powerful inhibitor of the Na^+^-K^+^ ATPase, has been used as a model of mania in rats [122]. Transgenic mice overexpressing GSK3 represent animal models of mania, too. They are characterized by increased locomotor activity, decreased habituation, and reduced brain weight [123]. It has been reported that lithium and the other mood stabilizer valproates protect the cells from the ouabain-induced apoptosis [124]. It is fascinating that the Na^+^-K^+^ ATPase has been demonstrated to be a link between oxidative damage and neuronal death reported in BD [125]. In a study published in 2012, the lithium-related enhancement of Na^+^-K^+^ ATPase activity related to a reduction of lipid peroxidation in bipolar patients [126]. Taking CREB into consideration, it is known that GSK3 inhibits its activity, while lithium does the opposite [127]. The CREB pathway is a key step of neuroprotection against OS [128]. Once again, the link between OS, GSK3, and BD is confirmed.

## 3. Concluding Remarks

In the light of what has been here reported, the main biological alterations of BD, pertaining to inflammation, oxidative stress, membrane ion channels, and circadian system, seem to be intertwined. The dysfunction of the GSK3 signalling pathway is involved in* all* the aforementioned “biological causes” of BD. In a complex scenario, it can be seen as the common denominator linking them all. Lithium inhibition of GSK3 could, at least in part, explain its positive effect on these biological dysfunctions and its superiority in terms of clinical efficacy. Deepening the knowledge on the molecular bases of BD is fundamental to identifying the biochemical pathways that must be targeted in order to provide patients with increasingly effective therapeutic tools against an invalidating disorder such as BD.

## References

[1] Rayasam G. V., Tulasi V. K., Sodhi R., Davis J. A., Ray A. (2009). Glycogen synthase kinase 3: more than a namesake. *British Journal of Pharmacology*.

[2] Ougolkov A. V., Billadeau D. D. (2008). Inhibition of glycogen synthase kinase-3. *Methods in Molecular Biology*.

[3] Steckert A. V., Valvassori S. S., Moretti M., Dal-Pizzol F., Quevedo J. (2010). Role of oxidative stress in the pathophysiology of bipolar disorder. *Neurochemical Research*.

[4] Wang Z., Ge Y., Bao H., Dworkin L., Peng A., Gong R. (2013). Redox-sensitive glycogen synthase kinase 3*β*-directed control of mitochondrial permeability transition: rheostatic regulation of acute kidney injury. *Free Radical Biology and Medicine*.

[5] Anderson G., Maes M. (2015). Bipolar disorder: role of immune-inflammatory cytokines, oxidative and nitrosative stress and tryptophan catabolites. *Current Psychiatry Reports*.

[6] Luca M., Prossimo G., Messina V., Luca A., Romeo S., Calandra C. (2013). Epidemiology and treatment of mood disorders in a day hospital setting from 1996 to 2007: an Italian study. *Neuropsychiatric Disease and Treatment*.

[7] Viswanath B., Jose S. P., Squassina A. (2015). Cellular models to study bipolar disorder: a systematic review. *Journal of Affective Disorders*.

[8] Wu D., Pan W. (2010). GSK3: a multifaceted kinase in Wnt signaling. *Trends in Biochemical Sciences*.

[9] Grimes C. A., Jope R. S. (2001). The multifaceted roles of glycogen synthase kinase 3*β* in cellular signaling. *Progress in Neurobiology*.

[10] Metcalfe C., Bienz M. (2011). Inhibition of GSK3 by Wnt signalling-two contrasting models. *Journal of Cell Science*.

[11] Sayas C. L., Ariaens A., Ponsioen B., Moolenaar W. H. (2006). GSK-3 is activated by the tyrosine kinase Pyk2 during LPA 1-mediated neurite retraction. *Molecular Biology of the Cell*.

[12] Medina M., Wandosell F. (2011). Deconstructing GSK-3: the fine regulation of its activity. *International Journal of Alzheimer's Disease*.

[13] Sutherland C. (2011). What are the bona fide GSK3 substrates?. *International Journal of Alzheimer's Disease*.

[14] Hur E.-M., Zhou F.-Q. (2010). GSK3 signalling in neural development. *Nature Reviews Neuroscience*.

[15] Salinas P. C., Hall A. C. (1999). Lithium and synaptic plasticity. *Bipolar Disorders*.

[16] Luo J. (2012). The role of GSK3beta in the development of the central nervous system. *Frontiers in Biology*.

[17] Cousin M. A., Smillie K. J. (2011). The role of GSK3 in presynaptic function. *International Journal of Alzheimer's Disease*.

[18] Wildburger N. C., Laezza F. (2012). Control of neuronal ion channel function by glycogen synthase kinase-3: new prospective for an old kinase. *Frontiers in Molecular Neuroscience*.

[19] Yeste-Velasco M., Folch J., Trullàs R. (2007). Glycogen synthase kinase-3 is involved in the regulation of the cell cycle in cerebellar granule cells. *Neuropharmacology*.

[20] Fiedorowicz M., Grieb P., Gonzalez-Quevedo A. (2012). Nitrooxidative stress and neurodegeneration. *Brain Damage—Bridging Between Basic Research and Clinics*.

[21] Wang C.-H., Wu S.-B., Wu Y.-T., Wei Y.-H. (2013). Oxidative stress response elicited by mitochondrial dysfunction: implication in the pathophysiology of aging. *Experimental Biology and Medicine*.

[22] Tanno M., Kuno A., Ishikawa S. (2014). Translocation of glycogen synthase kinase-3*β* (GSK-3*β*), a trigger of permeability transition, is kinase activity-dependent and mediated by interaction with voltage-dependent anion channel 2 (VDAC2). *The Journal of Biological Chemistry*.

[23] Dozza B., Smith M. A., Perry G., Tabaton M., Strocchi P. (2004). Regulation of glycogen synthase kinase-3*β* by products of lipid peroxidation in human neuroblastoma cells. *Journal of Neurochemistry*.

[24] Petit-Paitel A., Brau F., Cazareth J., Chabry J. (2009). Involvment of cytosolic and mitochondrial GSK-3*β* in mitochondrial dysfunction and neuronal cell death of MPTP/MPP+-treated neurons. *PLoS ONE*.

[25] King T. D., Bijur G. N., Jope R. S. (2001). Caspase-3 activation induced by inhibition of mitochondrial complex I is facilitated by glycogen synthase kinase-3*β* and attenuated by lithium. *Brain Research*.

[26] Bielecka A. M., Obuchowicz E. (2008). Antiapoptotic action of lithium and valproate. *Pharmacological Reports*.

[27] Maroni P., Bendinelli P., Zuccorononno C., Schiaffonati L., Piccoletti R. (2000). Cellular signalling after in vivo heat shock in the liver. *Cell Biology International*.

[28] Maroni P., Bendinelli P., Tiberio L., Rovetta F., Piccoletti R., Schiaffonati L. (2003). In vivo heat-shock response in the brain: signalling pathway and transcription factor activation. *Molecular Brain Research*.

[29] Meares G. P., Zmijewska A. A., Jope R. S. (2008). HSP105 interacts with GRP78 and GSK3 and promotes ER stress-induced caspase-3 activation. *Cellular Signalling*.

[30] Dou F., Chang X., Ma D. (2007). Hsp90 maintains the stability and function of the tau phosphorylating kinase GSK3*β*. *International Journal of Molecular Sciences*.

[31] Muller P., Ruckova E., Halada P. (2013). C-terminal phosphorylation of Hsp70 and Hsp90 regulates alternate binding to co-chaperones CHIP and HOP to determine cellular protein folding/degradation balances. *Oncogene*.

[32] Venè R., Cardinali B., Arena G. (2014). Glycogen synthase kinase 3 regulates cell death and survival signaling in tumor cells under redox stress. *Neoplasia*.

[33] Kim S., Joe Y., Kim H. J. (2015). Endoplasmic reticulum stress—induced IRE1*α* activation mediates cross-talk of GSK-3*β* and XBP-1 to regulate inflammatory cytokine production. *Journal of Immunology*.

[34] Biswas M., Chan J. Y. (2012). GSK3 negatively regulates stress response mediated by transcription factor Nrf1, resulting in increased neuronal apoptosis and neurodegeneration. *The FASEB Journal*.

[35] Venè R., Tosetti F., Lazo P. A. (2010). The role of glycogen synthase kinase-3 in the decision between cell survival and cell death. *Emerging Signaling Pathways in Tumor Biology*.

[36] Jacobs K. M., Bhave S. R., Ferraro D. J., Jaboin J. J., Hallahan D. E., Thotala D. (2012). GSK-3*β*: a bifunctional role in cell death pathways. *International Journal of Cell Biology*.

[37] Jope R. S., Yuskaitis C. J., Beurel E. (2007). Glycogen synthase kinase-3 (GSK3): inflammation, diseases, and therapeutics. *Neurochemical Research*.

[38] Barbosa I. G., Bauer M. E., MacHado-Vieira R., Teixeira A. L. (2014). Cytokines in bipolar disorder: paving the way for neuroprogression. *Neural Plasticity*.

[39] Leboyer M., Soreca I., Scott J. (2012). Can bipolar disorder be viewed as a multi-system inflammatory disease?. *Journal of Affective Disorders*.

[40] Beurel E., Jope R. S. (2015). Inflammation and lithium: clues to mechanisms contributing to suicide-linked traits. *Translational Psychiatry*.

[41] Kim H. K., Andreazza A. C., Elmi N., Chen W., Young L. T. (2016). Nod-like receptor pyrin containing 3 (NLRP3) in the post-mortem frontal cortex from patients with bipolar disorder: a potential mediator between mitochondria and immune-activation. *Journal of Psychiatric Research*.

[42] Wang L., Cheng B.-F., Yang H.-J., Wang M., Feng Z.-W. (2015). NF-*κ*B protects human neuroblastoma cells from nitric oxide-induced apoptosis through upregulating biglycan. *American Journal of Translational Research*.

[43] Berk M., Kapczinski F., Andreazza A. C. (2011). Pathways underlying neuroprogression in bipolar disorder: focus on inflammation, oxidative stress and neurotrophic factors. *Neuroscience and Biobehavioral Reviews*.

[44] Andreazza A. C., Kapczinski F., Kauer-Sant'Anna M. (2009). 3-Nitrotyrosine and glutathione antioxidant system in patients in the early and late stages of bipolar disorder. *Journal of Psychiatry and Neuroscience*.

[45] Akarsu S., Torun D., Erdem M., Kozan S., Akar H., Uzun O. (2015). Mitochondrial complex I and III mRNA levels in bipolar disorder. *Journal of Affective Disorders*.

[46] Callaly E., Walder K., Morris G. (2015). Mitochondrial dysfunction in the pathophysiology of bipolar disorder: effects of pharmacotherapy. *Mini-Reviews in Medicinal Chemistry*.

[47] Hayashi A., Kasahara T., Kametani M., Toyota T., Yoshikawa T., Kato T. (2009). Aberrant endoplasmic reticulum stress response in lymphoblastoid cells from patients with bipolar disorder. *International Journal of Neuropsychopharmacology*.

[48] Judy J. T., Zandi P. P. (2013). A review of potassium channels in bipolar disorder. *Frontiers in Genetics*.

[49] Imbrici P., Camerino D. C., Tricarico D. (2013). Major channels involved in neuropsychiatric disorders and therapeutic perspectives. *Frontiers in Genetics*.

[50] Kato T., Ishiwata M., Mori K. (2003). Mechanisms of altered Ca^2+^ signalling in transformed lymphoblastoid cells from patients with bipolar disorder. *International Journal of Neuropsychopharmacology*.

[51] Murray G., Harvey A. (2010). Circadian rhythms and sleep in bipolar disorder. *Bipolar Disorders*.

[52] Bellivier F., Geoffroy P.-A., Etain B., Scott J. (2015). Sleep- and circadian rhythm-associated pathways as therapeutic targets in bipolar disorder. *Expert Opinion on Therapeutic Targets*.

[53] Nováková M., Praško J., Látalová K., Sládek M., Sumová A. (2015). The circadian system of patients with bipolar disorder differs in episodes of mania and depression. *Bipolar Disorders*.

[54] Mansour H. A., Monk T. H., Nimgaonkar V. L. (2005). Circadian genes and bipolar disorder. *Annals of Medicine*.

[55] Nievergelt C. M., Kripke D. F., Barrett T. B. (2006). Suggestive evidence for association of the circadian genes PERIOD3 and ARNTL with bipolar disorder. *American Journal of Medical Genetics-Neuropsychiatric Genetics*.

[56] Cudney L. E., Sassi R. B., Behr G. A. (2014). Alterations in circadian rhythms are associated with increased lipid peroxidation in females with bipolar disorder. *International Journal of Neuropsychopharmacology*.

[57] Abreu T., Bragança M. (2015). The bipolarity of light and dark: a review on Bipolar Disorder and circadian cycles. *Journal of Affective Disorders*.

[58] Luca A., Luca M., Calandra C. (2013). Sleep disorders and depression: brief review of the literature, case report, and nonpharmacologic interventions for depression. *Clinical Interventions in Aging*.

[59] Bechtold D. A., Loudon A. S. I. (2013). Hypothalamic clocks and rhythms in feeding behaviour. *Trends in Neurosciences*.

[60] Benedetti F., Dallaspezia S. (2009). Melatonin, circadian rhythms, and the clock genes in bipolar disorder. *Current Psychiatry Reports*.

[61] Wirz-Justice A. (2003). Chronobiology and mood disorders. *Dialogues in Clinical Neuroscience*.

[62] Meares G. P., Jope R. S. (2007). Resolution of the nuclear localization mechanism of glycogen synthase kinase-3: functional effects in apoptosis. *The Journal of Biological Chemistry*.

[63] Wang M.-J., Huang H.-Y., Chen W.-F., Chang H.-F., Kuo J.-S. (2010). Glycogen synthase kinase-3*β* inactivation inhibits tumor necrosis factor-*α* production in microglia by modulating nuclear factor *κ*B and MLK3/JNK signaling cascades. *Journal of Neuroinflammation*.

[64] Beurel E., Jope R. S. (2006). The paradoxical pro- and anti-apoptotic actions of GSK3 in the intrinsic and extrinsic apoptosis signaling pathways. *Progress in Neurobiology*.

[65] Brooks M. M., Neelam S., Cammarata P. R. (2013). Lenticular mitoprotection. Part B: GSK-3*β* and regulation of mitochondrial permeability transition for lens epithelial cells in atmospheric oxygen. *Molecular Vision*.

[66] Chiara F., Gambalunga A., Sciacovelli M. (2012). Chemotherapeutic induction of mitochondrial oxidative stress activates GSK-3*α*/*β* and Bax, leading to permeability transition pore opening and tumor cell death. *Cell Death and Disease*.

[67] Dal Santo S., Stampfl H., Krasensky J. (2012). Stress-induced GSK3 regulates the redox stress response by phosphorylating glucose-6-phosphate dehydrogenase in Arabidopsis. *Plant Cell*.

[68] Sang M. S., Il J. C., Sang G. K. (2009). Resveratrol protects mitochondria against oxidative stress through AMP-activated protein kinase-mediated glycogen synthase kinase-3*β* inhibition downstream of poly(ADP-ribose) polymerase-LKB1 pathway. *Molecular Pharmacology*.

[69] Kitagishi Y., Kobayashi M., Kikuta K., Matsuda S. (2012). Roles of PI3K/AKT/GSK3/mTOR pathway in cell signaling of mental illnesses. *Depression Research and Treatment*.

[70] Tanno M., Miki T., Kuno A. (2011). GSK-3*β* translocates to mitochondria by oxidant stress in a kinase activity-dependent manner and contributes to lethal ROS production. *Circulation*.

[71] Uranga R. M., Katz S., Salvador G. A. (2013). Enhanced phosphatidylinositol 3-kinase (PI3K)/Akt signaling has pleiotropic targets in hippocampal neurons exposed to iron-induced oxidative stress. *Journal of Biological Chemistry*.

[72] Feng Y., Xia Y., Yu G. (2013). Cleavage of GSK-3*β* by calpain counteracts the inhibitory effect of Ser9 phosphorylation on GSK-3*β* activity induced by H_2_O_2_. *Journal of Neurochemistry*.

[73] Goñi-Oliver P., Lucas J. J., Avila J., Hernández F. (2007). N-terminal cleavage of GSK-3 by calpain: a new form of GSK-3 regulation. *Journal of Biological Chemistry*.

[74] Patel J. P., Frey B. N. (2015). Disruption in the blood-brain barrier: the missing link between brain and body inflammation in bipolar disorder?. *Neural Plasticity*.

[75] Lim J. C., Kania K. D., Wijesuriya H. (2008). Activation of *β*-catenin signalling by GSK-3 inhibition increases p-glycoprotein expression in brain endothelial cells. *Journal of Neurochemistry*.

[76] Ramirez S. H., Fan S., Zhang M. (2010). Inhibition of glycogen synthase kinase 3*β* (GSK3*β*) decreases inflammatory responses in brain endothelial cells. *American Journal of Pathology*.

[77] Jope R. S., Roh M.-S. (2006). Glycogen synthase kinase-3 (GSK3) in psychiatric disease and therapeutic interventions. *Current Drug Targets*.

[78] Karlsson J. O., Petersen A., Zetterberg M., Zetterberg H., Sjostrand J. (2005). Inhibition of glycogen synthase kinase (GSK-3) protects against oxidative stress and attenuates apoptosis in human lens epithelial cells and the mouse lens in organ culture. *Investigative Ophthalmology & Visual Science*.

[79] James T. F., Nenov M. N., Wildburger N. C. (2015). The Nav1.2 channel is regulated by GSK3. *Biochimica et Biophysica Acta—General Subjects*.

[80] Zhu L.-Q., Liu D., Hu J. (2010). GSK-3*β* inhibits presynaptic vesicle exocytosis by phosphorylating P/Q-type calcium channel and interrupting snare complex formation. *Journal of Neuroscience*.

[81] Shavkunov A. S., Wildburger N. C., Nenov M. N. (2013). The fibroblast growth factor 14·voltage-gated sodium channel complex is a new target of glycogen synthase kinase 3 (GSK3). *Journal of Biological Chemistry*.

[82] Chen P., Gu Z., Liu W., Yan Z. (2007). Glycogen synthase kinase 3 regulates N-methyl-D-aspartate receptor channel trafficking and function in cortical neurons. *Molecular Pharmacology*.

[83] Iitaka C., Miyazaki K., Akaike T., Ishida N. (2005). A role for glycogen synthase kinase-3*β* in the mammalian circadian clock. *Journal of Biological Chemistry*.

[84] Besing R. C., Paul J. R., Hablitz L. M. (2015). Circadian rhythmicity of active GSK3 isoforms modulates molecular clock gene rhythms in the suprachiasmatic nucleus. *Journal of Biological Rhythms*.

[85] Kaladchibachi S. A., Doble B., Anthopoulos N., Woodgett J. R., Manoukian A. S. (2007). Glycogen synthase kinase 3, circadian rhythms, and bipolar disorder: a molecular link in the therapeutic action of lithium. *Journal of Circadian Rhythms*.

[86] Li X., Liu M., Cai Z., Wang G., Li X. (2010). Regulation of glycogen synthase kinase-3 during bipolar mania treatment. *Bipolar Disorders*.

[87] Lavoie J., Hébert M., Beaulieu J.-M. (2014). Dlycogen synthase kinase-3 overexpression replicates electroretinogram anomalies of offspring at high genetic risk for schizophrenia and bipolar disorder. *Biological Psychiatry*.

[88] Li X., Jope R. S. (2010). Is glycogen synthase kinase-3 a central modulator in mood regulation. *Neuropsychopharmacology*.

[89] Benedetti F., Bernasconi A., Lorenzi C. (2004). A single nucleotide polymorphism in glycogen synthase kinase 3-*β* promoter gene influences onset of illness in patients affected by bipolar disorder. *Neuroscience Letters*.

[90] Benedetti F., Bollettini I., Barberi I. (2013). Lithium and GSK3-*β* promoter gene variants influence white matter microstructure in bipolar disorder. *Neuropsychopharmacology*.

[91] Jiménez E., Arias B., Mitjans M. (2013). Genetic variability at IMPA2, INPP1 and GSK3*β* increases the risk of suicidal behavior in bipolar patients. *European Neuropsychopharmacology*.

[92] Polter A., Beurel E., Yang S. (2010). Deficiency in the inhibitory serine-phosphorylation of glycogen synthase kinase-3 increases sensitivity to mood disturbances. *Neuropsychopharmacology*.

[93] Cade J. F. J. (2000). Lithium salts in the treatment of psychotic excitement. 1949. *Bulletin of the World Health Organization*.

[94] Schou M., Juel-nielsen N., Stromgren E., Voldby H. (1954). The treatment of manic psychoses by the administration of lithium salts. *Journal of Neurology, Neurosurgery, and Psychiatry*.

[95] Balon R. (2009). Old but still gold: lithium in stabilizing the mood. *Indian Journal of Psychiatry*.

[96] Freland L., Beaulieu J.-M. (2012). Inhibition of GSK3 by lithium, from single molecules to signaling networks. *Frontiers in Molecular Neuroscience*.

[97] Kishore B. K., Ecelbarger C. M. (2013). Lithium: a versatile tool for understanding renal physiology. *American Journal of Physiology—Renal Physiology*.

[98] de Sousa R. T., Zanetti M. V., Talib L. L. (2015). Lithium increases platelet serine-9 phosphorylated GSK-3*β* levels in drug-free bipolar disorder during depressive episodes. *Journal of Psychiatric Research*.

[99] Forlenza O. V., De-Paula V. J. R., Diniz B. S. O. (2014). Neuroprotective effects of lithium: implications for the treatment of Alzheimer's disease and related neurodegenerative disorders. *ACS Chemical Neuroscience*.

[100] de Sousa R. T., van de Bilt M. T., Diniz B. S. (2011). Lithium increases plasma brain-derived neurotrophic factor in acute bipolar mania: a preliminary 4-week study. *Neuroscience Letters*.

[101] Fukumoto T., Morinobu S., Okamoto Y., Kagaya A., Yamawaki S. (2001). Chronic lithium treatment increases the expression of brain-derived neurotrophic factor in the rat brain. *Psychopharmacology*.

[102] Cunha A. B. M., Frey B. N., Andreazza A. C. (2006). Serum brain-derived neurotrophic factor is decreased in bipolar disorder during depressive and manic episodes. *Neuroscience Letters*.

[103] Fernandes B. S., Gama C. S., Maria Ceresér K. (2011). Brain-derived neurotrophic factor as a state-marker of mood episodes in bipolar disorders: a systematic review and meta-regression analysis. *Journal of Psychiatric Research*.

[104] Machado-Vieira R., Andreazza A. C., Viale C. I. (2007). Oxidative stress parameters in unmedicated and treated bipolar subjects during initial manic episode: a possible role for lithium antioxidant effects. *Neuroscience Letters*.

[105] Khairova R., Pawar R., Salvadore G. (2012). Effects of lithium on oxidative stress parameters in healthy subjects. *Molecular Medicine Reports*.

[106] Maurer I. C., Schippel P., Volz H.-P. (2009). Lithium-induced enhancement of mitochondrial oxidative phosphorylation in human brain tissue. *Bipolar Disorders*.

[107] Machado-Vieira R., Manji H. K., Zarate C. A. (2009). The role of lithium in the treatment of bipolar disorder: convergent evidence for neurotrophic effects as a unifying hypothesis. *Bipolar Disorders*.

[108] Schäfer M., Goodenough S., Moosmann B., Behl C. (2004). Inhibition of glycogen synthase kinase 3*β* is involved in the resistance to oxidative stress in neuronal HT22 cells. *Brain Research*.

[109] Hokin-Neaverson M., Jefferson J. W. (1989). Deficient erythrocyte NaK-ATPase activity in different affective states in bipolar affective disorder and normalization by lithium therapy. *Neuropsychobiology*.

[110] de Lores Arnaiz Arnaiz G. R., Ordieres M. G. (2014). Brain Na^+^, K^+^-ATPase activity in aging and disease. *International Journal of Biomedical Science*.

[111] Berridge M. J. (2014). Calcium signalling and psychiatric disease: bipolar disorder and schizophrenia. *Cell and Tissue Research*.

[112] Mertens J., Wang Q.-W., Kim Y. (2015). Differential responses to lithium in hyperexcitable neurons from patients with bipolar disorder. *Nature*.

[113] Dubovsky S. L., Christiano J., Daniell L. C. (1989). Increased platelet intracellular calcium concentration in patients with bipolar affective disorders. *Archives of General Psychiatry*.

[114] Butler-Munro C., Coddington E. J., Shirley C. H., Heyward P. M. (2010). Lithium modulates cortical excitability in vitro. *Brain Research*.

[115] Yanagita T., Maruta T., Nemoto T. (2009). Chronic lithium treatment up-regulates cell surface NaV1.7 sodium channels via inhibition of glycogen synthase kinase-3 in adrenal chromaffin cells: enhancement of Na^+^ influx, Ca^2+^ influx and catecholamine secretion after lithium withdrawal. *Neuropharmacology*.

[116] Wallace J. (2014). Calcium dysregulation, and lithium treatment to forestall Alzheimer's disease-a merging of hypotheses. *Cell Calcium*.

[117] Palty R., Ohana E., Hershfinkel M. (2004). Lithium-calcium exchange is mediated by a distinct potassium-independent sodium-calcium exchanger. *The Journal of Biological Chemistry*.

[118] King T. D., Gandy J. C., Bijur G. N. (2006). The protein phosphatase-1/inhibitor-2 complex differentially regulates GSK3 dephosphorylation and increases sarcoplasmic/endoplasmic reticulum calcium ATPase 2 levels. *Experimental Cell Research*.

[119] Lin Y.-F., Huang M.-C., Liu H.-C. (2013). Glycogen synthase kinase 3*β* gene polymorphisms may be associated with bipolar I disorder and the therapeutic response to lithium. *Journal of Affective Disorders*.

[120] Rybakowski J. K., Abramowicz M., Szczepankiewicz A., Michalak M., Hauser J., Czekalski S. (2013). The association of glycogen synthase kinase-3beta (GSK-3*β*) gene polymorphism with kidney function in long-term lithium-treated bipolar patients. *International Journal of Bipolar Disorders*.

[121] Alda M. (2015). Lithium in the treatment of bipolar disorder: pharmacology and pharmacogenetics. *Molecular Psychiatry*.

[122] El-Mallakh R. S., El-Masri M. A., O'Malley Huff M., Li X.-P., Decker S., Levy R. S. (2003). Intracerebroventricular administration of ouabain as a model of mania in rats. *Bipolar Disorders*.

[123] Prickaerts J., Moechars D., Cryns K. (2006). Transgenic mice overexpressing glycogen synthase kinase 3*β*: a putative model of hyperactivity and mania. *Journal of Neuroscience*.

[124] Hennion J. P., Adnan El-Masri M., Huff M. O., El-Mallakh R. S. (2002). Evaluation of neuroprotection by lithium and valproic acid against ouabain-induced cell damage. *Bipolar Disorders*.

[125] Valvassori S. S., Resende W. R., Lopes-Borges J. (2015). Effects of mood stabilizers on oxidative stress-induced cell death signaling pathways in the brains of rats subjected to the ouabain-induced animal model of mania. Mood stabilizers exert protective effects against ouabain-induced activation of the cell death pathway. *Journal of Psychiatric Research*.

[126] Banerjee U., Dasgupta A., Rout J. K., Singh O. P. (2012). Effects of lithium therapy on Na +-K +-ATPase activity and lipid peroxidation in bipolar disorder. *Progress in Neuro-Psychopharmacology and Biological Psychiatry*.

[127] Grimes C. A., Jope R. S. (2001). Creb DNA binding activity is inhibited by glycogen synthase kinase-3*β* and facilitated by lithium. *Journal of Neurochemistry*.

[128] Lee B., Cao R., Choi Y.-S. (2009). The CREB/CRE transcriptional pathway: protection against oxidative stress-mediated neuronal cell death. *Journal of Neurochemistry*.

